# Achieving thermostability of a phytase with resistance up to 100 °C

**DOI:** 10.1016/j.jbc.2024.107992

**Published:** 2024-11-14

**Authors:** Tao Tu, Qian Wang, Ruyue Dong, Xiaoqing Liu, Leena Penttinen, Nina Hakulinen, Jian Tian, Wei Zhang, Yaru Wang, Huiying Luo, Bin Yao, Huoqing Huang

**Affiliations:** 1 State Key Laboratory of Animal Nutrition and Feeding, Institute of Animal Sciences, Chinese Academy of Agricultural Sciences, Beijing, China; 2 Biotechnology Research Institute, Chinese Academy of Agricultural Sciences, Beijing, China; 3 Department of Chemistry, University of Eastern Finland, Joensuu Campus, Joensuu, Finland

**Keywords:** hyperthermophilic, phytase, thermostability, boiling point, rational design

## Abstract

The development of enzymes with high-temperature resistance up to 100 °C is of significant and practical value in advancing the sustainability of industrial production. Phytase, a crucial enzyme in feed industrial applications, encounters challenges due to its limited heat resistance. Herein, we employed rational design strategies involving the introduction of disulfide bonds, free energy calculation, and B-factor analysis based on the crystal structure of phytase APPAmut4 (1.90 Å), a variant with enhanced expression levels derived from *Yersinia intermedia*, to improve its thermostability. Among the 144 variants experimentally verified, 29 exhibited significantly improved thermostability with higher *t*_1/2_ values at 65 °C. Further combination and superposition led to APPAmut9 with an accumulation of five additional pairs of disulfide bonds and six single-point mutation sites, leading to an enhancement in its thermostability with a *t*_1/2_ value of 256.7 min at 65 °C, which was more than 75-fold higher than that of APPAmut4 (3.4 min). APPAmut9 exhibited a *T*_50_ value of 96 °C, representing a substantial increase of 40.9 °C compared to APPAmut4. Notably, approximately 70% of enzyme activity remained intact after exposure to boiling water at 100 °C for a holding period of 5 min. Significantly, these advantageous modifications were strategically positioned away from the catalytic pocket where enzymatic reactions occur to ensure minimal compromise on catalytic efficiency between APPAmut9 (11,500 ± 1100/mM/s) and APPAmut4 (12,300 ± 1600/mM/s). This study demonstrates the feasibility of engineering phytases with resistance to boiling using rational design strategies.

Enzymes are favored over chemical catalysts due to their exceptional specificity, environmental compatibility, and catalytic efficiency, making them extensively employed in diverse large-scale industrial applications (1). However, the effectiveness of enzymes is constrained by their susceptibility to harsh production conditions such as high temperatures or exposure to organic solvents or proteases (2, 3). In particular, stringent requirements are often imposed by industrial production conditions. For instance, in the feed pelletizing process, the addition of feed enzymes requires an instantaneous high temperature treatment (70–95 °C) achieved through steam input and friction in the film press (4, 5). This technique not only mitigates dust-related issues and enhances livestock consumption of the feed but also plays a crucial role in boosting animals’ resistance to African swine fever virus or harmful bacteria (such as *Salmonella* spp.). The thermostability of enzymes is crucial in assessing the practicality of utilizing industrial enzyme preparations, due to the facilitation of accelerated reaction processes, increased enzyme reusability, and reduced production costs by robust enzymes (3). Unfortunately, the majority of natural enzymes are susceptible to denaturation at elevated temperatures (6), emphasizing the essential need for enhancing enzyme stability to preserve their functionality. Consequently, elucidating the mechanisms underlying enzyme stability has emerged as a prominent subject in the field of protein engineering.

Numerous studies have demonstrated that factors such as hydrogen bonds, salt bonds, and hydrophobic interactions exert a profound influence on enzyme stability (7). However, due to the intricate and multifaceted nature of these factors, reaching a definitive conclusion regarding the underlying mechanism governing enzyme stability remains elusive. Diverse strategies with varying degrees of success have been employed in designing approaches aimed at augmenting stability (8, 9, 10, 11), and site-specific mutation studies have shown potential for enhancing enzyme thermostability within a range of 2 to 15 °C. Importantly, a limited number of this approach has demonstrated an even greater degree of enhancement (>20 °C) (12). This suggests that the challenge of acquiring extremely thermophilic enzymes or achieving stability at boiling point through protein engineering remains substantial. Two notable successful cases include the development of a thermostable variant of a thermolysin-like protease capable of functioning at 100 °C, achieved by substituting residues at equivalent positions in its naturally occurring and well-known counterpart, thermolysin (13). Additionally, a computational method known as framework for rapid enzyme stabilization was utilized to generate an alcohol dehydrogenase variant with a *T*_m_ of 94 °C (14). These successful examples have demonstrated that current advancements in enzyme engineering requires exploring the intricate relationship between enzyme structure and function through innovative engineering approaches to develop tailored enzymes suitable for specific industrial applications (15).

Phytic acid serves as the primary reservoir of phosphorus in cereals, oilseeds, beans, and other plant tissues (16). Recognized as a prominent antinutrient factor in feed, phytic acid possesses an exceptionally high negative charge and exhibits robust chelating properties. Consequently, it effectively complexes with polyvalent metal cations, sugars, and proteins, thereby influencing mineral availability and hindering the hydrolytic absorption of nutrients such as sugars and proteins (17). The consumption of diets abundant in phytate significantly diminishes the absorption of iron, zinc, calcium, and magnesium compared to those with low phytate content. Particularly prevalent in low-income countries, malabsorption resulting from diets abundant in phytic acid constitutes a major contributor to iron and zinc deficiencies (16). Phytase has been authorized as a feed additive for over 2 decades and is widely recognized in the market as the predominant feed enzyme, constituting 60% of the entire industry (5). By hydrolyzing phytate—a compound that inhibits nutrient absorption found in animal feed—into inorganic phosphoric acid, it effectively reduces phosphorus excretion while enhancing mineral and protein digestion and absorption (18). As a result, this enzymatic process significantly improves animal production performance. However, the issue of heat resistance has emerged as a critical bottleneck hindering the industrial application of phytase due to the technical requirement of instantaneous high temperatures during feed pelletizing processes.

The initial generation of phytase derived from *Aspergillus niger* exhibits limited thermostability and high activity at a neutral pH (19). For instance, the *A*. *niger* phytase PhyA demonstrates significantly higher specific activity than the *Aspergillus fumigatus* phytase (102.5 U/mg *versus* 26.5 U/mg), but undergoes a 70 to 80% reduction in enzymatic activity under feed pelleting conditions (20). Subsequent studies have primarily focused on bacterial phytases, particularly those from *Escherichia coli*. The subsequent version of the enzyme exhibits enhanced specific activity and substrate affinity, while demonstrating superior performance in acidic conditions. Importantly, it has been observed that loop residues in close proximity to the substrate play a significant role in the activity of *E*. *coli* phytase, as evidenced by results from alanine and glycine scanning mutagenesis, which led to a reduction of over 90% in activity compared to the WT enzyme (21). Through protein engineering, the third generation of phytases has been developed with improved heat resistance capabilities. The variant AppA-Q258N/Q349N was generated through *N*-glycosylation modification, resulting in a 40% increase in residual activity after exposure to 85 °C for 10 min (22). It has been demonstrated that the C-terminal region of *E*. *coli* phytase plays a crucial role in thermostability, as evidenced by a deletion variant exhibiting approximately 39% higher residual activity than the WT following exposure to 80 °C for 10 min (23). A comparative analysis was conducted to evaluate the thermostability of four commercially available phytase products, namely Quantum Blue G from AB Vista, Ronozyme Hi Phos GT from DSM Nutritional Products, Axtra Phy TPT from Dupont, and Microtech 5000 Plus from Guangdong Vtr Bio-Tech Co., Ltd (24). This investigation revealed a consistent reduction in residual activity with increasing temperature, that is, a decline of 1.9% residual activity for every 1 °C rise in temperature within the range of 65 to 95 °C. The top performing enzymes exhibited less than 35% residual activity at 95 °C. Therefore, despite substantial advancements in understanding the characteristics of phytases, there remains a knowledge gap regarding the optimal enzyme formulations required for practical implementation.

The phytase APPA derived from *Yersinia intermedia* exhibits exceptional overall properties, including a high specific activity (3960 U/mg), excellent pH stability within the range of pH 2.5 to 10.0, and resistance to pepsin and trypsin digestion at 37 °C for 2 h, making it highly suitable for application in the feed industry (25). In this study, a variant named APPAmut4 was successfully obtained with significantly enhanced expression levels through directed evolution techniques applied to APPA, effectively reducing the production cost associated with this enzyme. To address the primary challenge of poor thermostability in APPAmut4 that limits its applicability in the feed industry, our study initially determined its crystal structure. This provided a solid theoretical foundation for rational design aimed at improving its thermostability. Building upon these findings, various strategies—including disulfide bond introduction, energy calculation, and B-factor analysis—were implemented to enhance the thermostability of APPAmut4. As a result of these efforts, an exceptional super phytase capable of withstanding temperatures up to 100 °C without any loss in catalytic efficiency was successfully developed. Our study demonstrates the feasibility of engineering phytases with resistance to boiling through rational design strategies and expands the repertoire of applicable phytases for industrial feed production.

## Result

### Characterization of APPAmut4

The *Y*. *intermedia* phytase APPA shows great potential for application in the feed industry due to its desirable properties (25). However, it falls short of meeting industrial production requirements in terms of thermostability and therefore necessitates improvement. In this study, we initially employed a random mutagenesis approach to modify the characteristics of APPA. Approximately, 8000 colonies were screened from the randomly generated variant library, with around 5000 colonies demonstrating phytase activity. To identify variants with improved thermostability, the supernatant from the 96-well plates was incubated at 65 °C for 5 min during the initial screening. Among these variants, a clear increase in residual activity compared to that of APPA was observed for a variant with four mutations (APPA_Y51E/V86D/A323L/M359L, designated as APPAmut4). However, further verification through shaking flask fermentation revealed a progressive increase in expression levels of both APPA and APPAmut4 following induction, with the expression level of APPAmut4 surpassing that of APPA. Upon quantitative analysis, it was determined that APPAmut4 exhibited comparable enzymatic properties to those of APPA. This suggests that the initial screening results may be attributed to higher protein expression of APPAmut4. The APPAmut4 exhibited optimal functionality at 55 °C, maintaining over half of its activity within the temperature range of 35 to 60 °C but becoming nearly inactive above 65 °C. Below 50 °C, the enzyme demonstrated stability; however, it experienced 45% decline in activity after a 2-min incubation and retained less than 10% activity following a 15-min incubation at 65 °C. Its half-life *t*_1/2_ value at 65 °C was approximately 3.4 min. Additionally, the *T*_50_ value was determined to be around 55.1 °C. The melting temperature, *T*_m_, of the APPAmut4 (58.2 ± 0.9 °C), corresponding to the temperature at which a decrease in catalytic activity is observed, was found to be comparable to that of APPA (58.1 ± 0.9 °C). Considering the importance and relevance of high-level production of heterologous proteins in both basic research and industrial applications, APPAmut4 served as an ideal candidate for enhancing its thermostability through the utilization of a complementary structure-based computational approach.

### Crystal structure of APPAmut4

**Figure 1:**
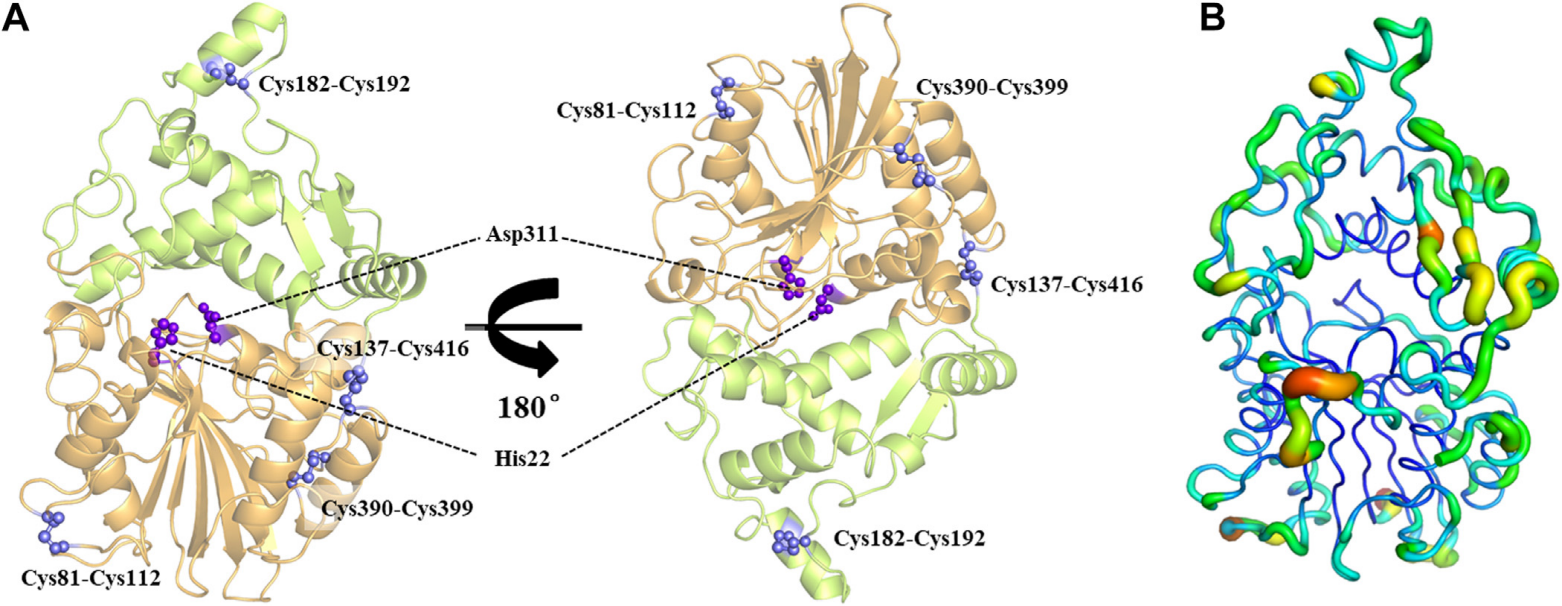
**The overall structure of APPAmut4.***A*, the *cartoon representation* of the structure depicts the α/β- and α-domains in *bright orange* and *limon*, respectively. The conserved catalytic residues and cysteine are appropriately labeled and visually presented as a ball-and-stick model in *purple blue* and *slate*. *B*, the B-factor visualization of APPAmut4 illustrates the local structural rigidity, ranging from *thin blue line* (value of 0.0) to *thick red* line (value of 4.0) indicating flexibility.

A prerequisite for the computational-guided engineering of enzymes is the availability of a high-quality crystal structure of APPAmut4 (14). Therefore, we crystallized APPAmut4 (20 mg/ml) to determine its structural basis for the observed differences compared with the WT, resulting in crystals belonging to the orthorhombic space group *P*2_1_2_1_2_1_. The apo-form crystal structure of APPAmut4 (PDB ID: 8XM1) was determined at a resolution of 1.89 Å, revealing a continuous polypeptide chain tracing from residue Gly10 to Ile418 within the asymmetric unit. To further characterize this structure, a comparison was conducted using the DALI online website (http://ekhidna2.biocenter.helsinki.fi/dali/). APPAmut4 exhibited a 1.7 Å RMSD for Cα atoms when compared with *E*. *coli* periplasmic phytase (PDB ID: 7Z1J), revealing no discernible conformational disparities between the two structures. This confirms that APPAmut4 adheres to the typical characteristics of a 1d-6-phytase, as observed in clade 2 of the histidine phosphatase family, which is primarily composed of α/β and α-domains (Fig. 1). The active site within the two domains typically consists of two conserved motifs: R*H*GXRXP in the N-terminal and H*D* in the C-terminal, with His and Asp serving as catalytic residues. This leads to the identification of the catalytic nucleophile His22 and proton donor Asp311 in the catalytic pocket (26). B-factors analysis indicates that the α/β domain demonstrates relatively lower flexibility than the α-domain, particularly in the β-sheet region, suggesting a higher degree of rigidity. The N- and C-terminal regions, along with loop regions (residue 76–86, 119–128, 138–144, 235–246, 348–351, and 392–395), display flexibility which may negatively impact protein stability due to their limited interactions with other residues and disruption of noncovalent connections within complex networks (27). Furthermore, four conserved disulfide bridges involving cysteine (Cys81-Cys112, Cys137-Cys416, Cys182-Cys192, and Cys390-Cys399) are present to enhance enzyme structure stabilization (9).

### Complementary predictions based on structured information

**Figure 2:**
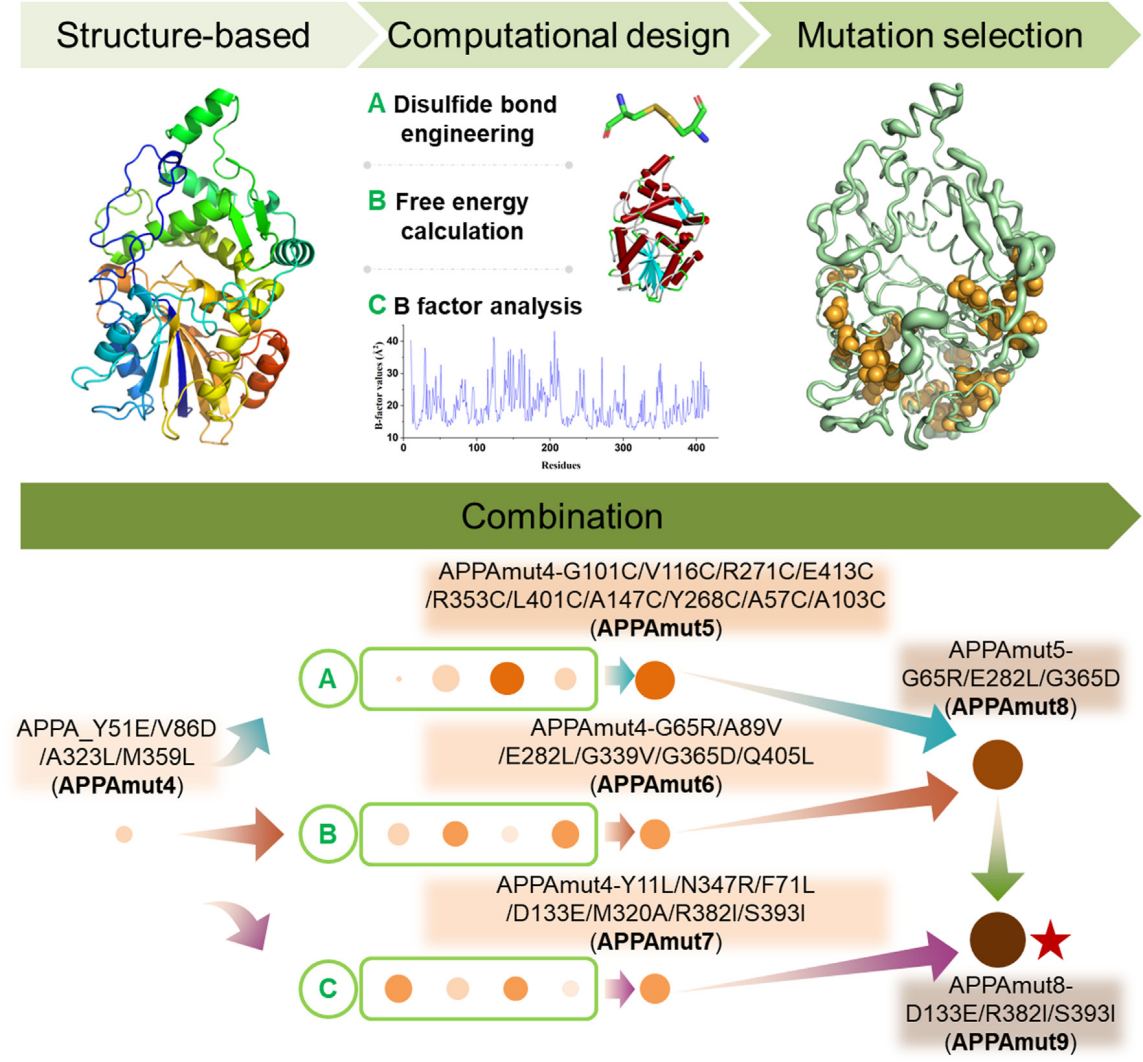
**Schematic representation of computational design based on the structural basis of APPAmut4.** Based on the crystal structure of phytase APPAmut4, three rational design strategies were implemented to enhance its thermostability, including the introduction of disulfide bonds (strategy *A*), energy calculations (strategy *B*), and B-factor analysis (strategy *C*). For strategy *A*, a total of 95 variants were designed, from which 19 were selected for experimental verification. Among these, seven variants demonstrated significantly improved thermostability with residual activity exceeding 50% after exposure to heat at 65 °C for 2 min. The optimal variant, APPAmut5, was engineered through the incorporation of beneficial mutations, leading to the formation of an additional five sets of disulfide bonds. Strategy *B* involved the design of a total of 1091 variants, from which 105 were selected for experimental verification. Ultimately, 15 positive variants with mutations at nine residues were identified, leading to the development of APPAmut6, which incorporates six mutations. Strategy *C* entailed meticulous selection and organization of 305 residues within APPAmut4, resulting in identification and experimental validation of 20 variants. This led to discovery of seven variants exhibiting residual enzyme activity surpassing that of APPAmut4. These mutation sites were combined and further screened to obtain the optimal variant APPAmut7, comprising six mutation sites. Subsequently, the nine advantageous sites identified by strategy *B* were overlaid onto APPAmut5, resulting in the accumulation of three mutation sites and the creation of APPAmut8. Based on APPAmut8, beneficial mutation sites obtained through strategy *C* were iteratively combined to successfully identify the most promising variant named as APPAmut9. The size of the screening circle indicates the residual enzymatic activity following heat treatment, with *larger circles* corresponding to higher levels of activity. The color intensity of the screening circle reflects the enzyme’s heat resistance temperature, with darker hues indicating greater thermostability.

In recent years, the advancement in structural biology and bioinformatics has greatly facilitated the widespread application of computational design methods that utilize protein structure and sequence information to enhance protein thermostability (28). Drawing on the structural basis of APPAmut4, three rational design strategies were employed to investigate its molecular design for enhanced thermostability (Fig. 2). First, APPAmut4 was rationally designed based on the introduction strategy of disulfide bonds, as this approach has demonstrated high effectiveness in enhancing enzyme stability (29). This immobilizes the enzyme structure and stabilizes its active conformation. It is worth noting, however, that not all introductions of disulfide bonds lead to enhanced stability. Therefore, we integrated the analysis results from Disulfide by Design and Discovery Studio to conduct precise engineering designs for introducing disulfide bonds into APPAmut4. Second, APPAmut4 was designed based on a rational strategy for calculation of free energy, with the unfolding free energy (ΔG) serving as a crucial parameter reflecting the thermodynamic stability of proteins. Different algorithms have distinct emphases and calculation methods, resulting in significant variations in prediction outcomes and success rates (30). Thus, to enhance the positive rate, FoldX, DeepDDG, CUPSAT, and HotSpot Wizard 3.0 were employed to analyze APPAmut4. The results were statistically summarized, and an intersection of different methods was utilized to construct a virtual variant library. Third, APPAmut4 was designed using a rational approach based on B-factor analysis strategy. The B-factor serves as a measure of the uncertainty of atomic position, reflecting the “diffusion” of atomic electron density within the crystal, effectively mirroring the conformational state of the protein molecule in the crystal (31). Higher B-factors correspond to increased flexibility and conformational instability in specific regions. Consequently, residues with elevated values were identified as mutation hotspots through meticulous analysis of their respective B-factors. Finally, all positively mutated variants obtained through the three strategies were sequentially incorporated to identify a variant with superior thermostability.

### Rational design of thermostability of APPAmut4 based on disulfide bond introduction strategy

**Figure 3:**
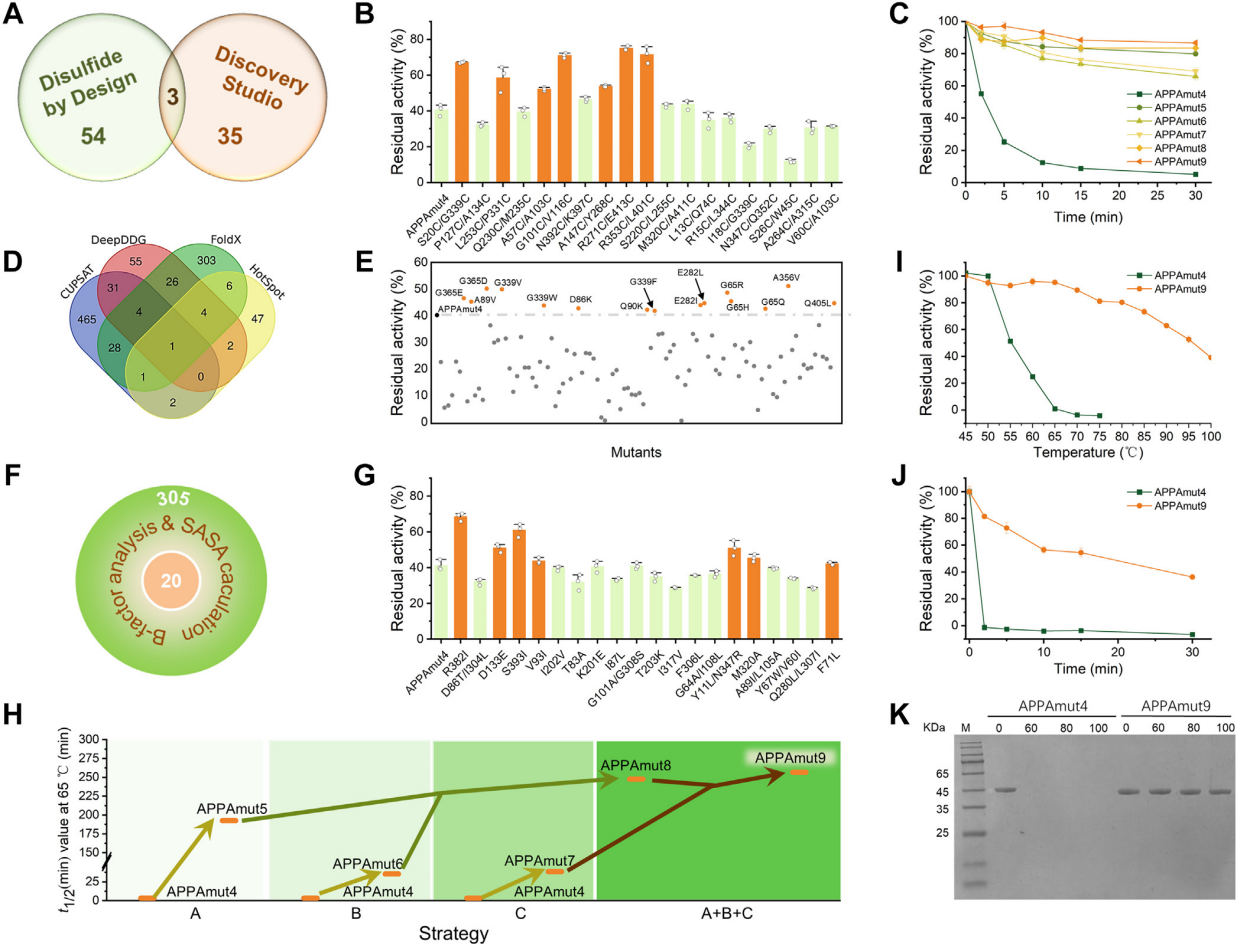
The enhancement of thermostability in APPAmut4. *A*, the rational design of thermostability in APPAmut4 through the strategic introduction of disulfide bonds (*A*), energy calculations (*D*), and B-factor analysis (*F*). The screening of selected variants undergoes experimental verification to strategically introduce disulfide bonds (*B*), energy calculations (*E*), and B-factor analysis (*G*). The residual enzyme activity of APPAmut4 and its variants were evaluated following a 2-min heat treatment at 65 °C. *C*, thermal inactivation curves at 65 °C for APPAmut4 and its variants, including APPAmut5, APPAmut6, APPAmut7, APPAmut8, and APPAmut9. The enzyme solution was suitably diluted and incubated at 65 °C for various time intervals, followed by measurement of the corresponding activity under standardized conditions. The initial activity served as the reference point (100%). *H*, the thermostability of APPAmut4 was significantly improved through the implementation of a multistrategy combination approach. *I*, the residual enzyme activity of APPAmut4 and its multistrategy combination variant APPAmut9 at different temperatures ranging from 45 to 100 °C over a period of 30 min. *J*, the thermal inactivation kinetics of APPAmut4 and APPAmut9 at 100 °C. The enzyme solution was appropriately diluted and incubated at 100 °C for various durations, followed by measurement of the corresponding activity under standardized conditions. *K*, the difference in protein content between APPAmut4 and APPAmut9 following treatment at high temperatures. The purified APPAmut4 and APPAmut9 proteins were exposed to 60 °C, 80 °C, and 100 °C for 10 min, followed by centrifugation at 12000 rpm for 10 min. The resulting supernatant was utilized for SDS-PAGE analysis.

The careful selection of mutation sites for introducing disulfide bonds is a critical factor in improving enzyme thermostability. Herein, we evaluated the strategy for introducing disulfide bonds by analyzing the design outcomes from Disulfide by Design (57 variants) and Discovery Studio 2017 (38 variants) (Fig. 3*A*). Following a comprehensive evaluation, the top 15 candidates from each strategy were chosen as potential variants. Variants H22C/R96C and D311C/T334C, which involved catalytic residues His22 and Asp311, were excluded to ensure enzyme functionality. Our analysis revealed the presence of four conserved disulfide bonds in APPAmut4 itself, eliminating the need for additional construction efforts. Furthermore, variants A147C/Y268C, A264C/A315C, and S26C/W45C consistently emerged in the analysis results of both software programs. In summary, a total of 19 variants were generated for experimental verification (Fig. 3*B*).

Of these 19 variants, seven variants exhibited significantly enhanced thermostability with residual activity greater than 50% after heat treatments at 65 °C for 2 min. The *t*_1/2_ value at 65 °C increased to varying degrees (5.5–18.6 min) for these seven variants compared to APPAmut4 (3.4 min). Among them, the variant R353C/L401C displayed the highest thermostability, with its *t*_1/2_ value approximately 5 times longer than that of APPAmut4. To further enhance the thermostability, the aforementioned seven beneficial mutations were combined, resulting in an improved thermostability achieved through additional formation of disulfide bonds. The optimal variant, designated as APPAmut5, contained additional five sets of disulfide bonds: G101C/V116C, R271C/E413C, R353C/L401C, A147C/Y268C, and A57C/A103C. Collectively, the APPAmut5 exhibited a high level of stability at 65 °C, with only approximately 20% enzyme activity lost after a 30-min heat treatment period (Fig. 3*C*). The calculated *t*_1/2_ value at 65 °C reached a duration of 192.5 min, approximately 56.6 times higher than that observed for APPAmut4. The *T*_m_ of APPAmut5 was elevated by 14.3 °C compared to APPAmut4, indicating that APPAmut5 has a higher unfolding temperature. Disulfide bonds play a crucial role in enhancing the reversible unfolding process of proteins by reducing their conformational entropy during unfolding (32). These covalent bonds create a higher free energy barrier for protein unfolding than noncovalent interactions like hydrogen bonding and hydrophobic interactions (33). Consequently, manipulating disulfide bonds offers an effective approach to improve protein thermostability, including that of phytase, as demonstrated in the present study.

### Rational design of thermostability of APPAmut4 based on free energy calculation strategy

A variety of algorithms are utilized to guide protein stability modification by predicting changes in ΔΔG (34). However, different algorithms have distinct emphases and calculation methods, leading to substantial variations in prediction outcomes and success rates (30). Therefore, four algorithms were employed to rationally design thermostable variants of APPAmut4 in order to increase the positive rate. The biodesign software FoldX was employed to conduct PositionScan and fully saturate residues at all sites in APPAmut4, resulting in the calculation of ΔΔG. This value represents the difference between the free folding energy of the variant and that of the WT, indicating increased stability when ΔΔG <0. Standard settings were applied and each calculation was repeated three times for improved averaging. Variants with ΔΔG <−1 kJ/mol were selected as candidates to enhance positive outcomes, yielding a total of 373 variants for further analysis. DeepDDG provides the capability to perform site-specific mutation calculations for proteins or full residue site saturation mutations; this study opted for the latter approach to analyze APPAmut4. When ΔΔG >0 in the output results, it signifies that the variant is more stable than the WT. A total of 123 variants with ΔΔG >1 kcal/mol were identified as potential candidates. The CUPSAT algorithm was utilized to conduct saturation mutation on all residues of APPAmut4, with *thermal* selected as the parameter for the Experimental method. The output of this method includes both predicted ΔΔG and *torsion* values. In this study, emphasis is placed on designing phytase based on energy calculations; therefore, only the predicted ΔΔG values are taken into consideration while *torsion* is disregarded. A total of 532 variants with ΔΔG >2 kcal/mol were selected as potential candidates. In HotSpot Wizard 3.0, a total of 63 candidate variants were discovered through the combination of the structural design module, which yielded 22 variants, and the sequence design module, which predicted 45 variants, with an overlap of four variants. The emphasis on computational analysis varies across these algorithms, resulting in significantly divergent outcomes. To streamline experiments and enhance success rates, a further analysis of the results is conducted, focusing on selecting the intersection between different methods for experimental verification. As shown in Fig. 3*D*, the intersection sets of four methods exhibited only one variant, while those of three distinct methods contained a total of nine variants, and the intersection sets of two methods yielded 95 variants. These 105 selected variants were subjected to experimental verification.

A total of 15 positive variants with mutations at nine amino acid residues were obtained (Fig. 3*E*). The *t*_1/2_ values of these nine positive variants measured at 65 °C exceeded that of APPAmut4 (3.4 min) and ranged between 3.8 to 9.1 min. By combining different mutation sites, we ultimately obtained APPAmut6 with six mutations: G65R, A89V, E282L, G339V, G365D, and Q405L. Following heat treatment at 65 °C for 15 min, the residual enzyme activity of APPAmut6 reached a level of 70%, while APPAmut4 exhibited nearly complete inactivity with a loss of over 90% in enzyme activity. The synergistic effects among multiple sites resulted in a 10-fold increase in the *t*_1/2_ value for APPAmut6 at 65 °C compared to that of APPAmut4, reaching 35.5 min (Fig. 3*C*). Furthermore, the *T*_m_ of APPAmut6 showed an increase of 3.5 °C compared to that of APPAmut4.

It is noteworthy that among the four algorithms employed, only one variant G365E was consistently identified by all, exhibiting a *t*_1/2_ value at 65 °C of approximately 0.5 min higher than APPAmut4 with an accuracy of 100%. By intersecting three algorithms, a total of nine predicted variants were obtained, wherein G365D and A89V displayed enhanced thermostability with an accuracy rate of 22.2%. The intersection of two algorithms yielded 95 variants, out of which 12 positive variants were identified with an accuracy rate of 12.6%. Individual algorithm performances resulted in accuracy rates of 3.2% (FoldX), 1.3% (CUPSAT), 7.3% (DeepDDG), and 9.5% (HotSpot Wizard 3.0). Consequently, employing a combination of diverse algorithms for enzyme stability design significantly enhances the prediction success rate compared to relying on a single algorithm.

### Rational design of thermostability of APPAmut4 based on B-factor analysis strategy

In our previous study, we successfully developed a design methodology to enhance enzyme thermostability by employing an analysis strategy based on B-factors (35). Initially, we meticulously selected and organized 305 amino acids within a 20 Å range encompassing the catalytic residues of APPAmut4, taking into consideration their respective B-factor values (Fig. 3*F*). Subsequently, we performed calculations to determine their solvent-accessible surface areas to identify residues with higher ratios of solvent exposure for further comprehensive analysis. Finally, through integration of conservative site analysis and meticulous design of local interaction networks, 20 variants were identified that were subsequently experimentally validated.

As shown in Fig. 3*G*, variants exhibited residual enzyme activities surpassing that of APPAmut4 after a 2-min heat treatment at 65 °C, indicating enhanced thermostability. These seven variants demonstrated higher *t*_1/2_ values compared to APPAmut4 at 65 °C. Specifically, the *t*_1/2_ value of S393I at 65 °C was measured as 7.5 min, which is approximately 2.2 times longer than that of APPAmut4 (3.4 min). Following closely behind is F71L with a *t*_1/2_ value of 7.0 min, representing a 2-fold increase compared to APPAmut4. Subsequently, these mutation sites were combined and further screened to obtain the optimal variant APPAmut7 comprising six mutation sites: Y11L/N347R, F71L, D133E, M320A, R382I, and S393I. Remarkably, the *t*_1/2_ value of APPAmut7 at 65 °C reached a duration of 38.7 min which is 11.4 times longer than that of APPAmut4 (Fig. 3*C*). In comparison with the *T*_m_ of APPAmut4, the *T*_m_ of the APPAmut7 showed increases by 3.9 °C.

Numerous studies have consistently demonstrated the prevalence of noncovalent interaction forces in proteins derived from thermophilic organisms (36). These interactions among residues play a crucial role in augmenting the energy barrier during thermal denaturation, thereby preserving the correct conformation and ensuring enhanced structural rigidity. Consequently, proteins exhibit remarkable thermostability. In our study, we aimed to enhance the thermostability of phytase by strategically substituting flexible amino acids within the APPAmut4 structure. This approach effectively bolstered structural rigidity, curtailed conformational flexibility, and fostered increased interdomain interactions to further stabilize unstable domains.

### Combining mutations

The three aforementioned strategies were implemented to enhance the thermostability of APPAmut4, resulting in the creation of the combined variants APPAmut5, APPAmut6, and APPAmut7. These variants exhibited a significant improvement in thermostability compared to APPAmut4 (Fig. 3*H*). The diverse strategies operate on distinct principles and have the potential to complement each other, thereby enhancing their industrial applicability. Subsequently, we explored interstrategy combination mutations with the objective of obtaining phytase variants that demonstrate superior thermostability. The energy calculation strategy identified nine advantageous sites, which were subsequently overlaid onto APPAmut5. Through a process of screening and combination based on residual enzyme activity after exposure to 90 °C for 5 min, variant APPAmut8 was successfully obtained. Building upon the foundation laid by APPAmut5, three mutation sites (G65R, E282L, and G365D) were accumulated. Notably, the *t*_1/2_ value of APPAmut8 at 65 °C was further extended to 247.6 min, representing a significant increase of 55.1 min compared to that of APPAmut5 and an enhancement by a factor of 72.8-fold when compared to APPAmut4 (Fig. 3*C*). The *T*_m_ of APPAmut8 was found to increase by 4.9 °C, reaching a value of 77.4 ± 1.2 °C. Subsequently, the beneficial mutation sites obtained through the B-factor analysis strategy were iteratively combined based on APPAmut8. Through meticulous screening of overlapping mutation sites, the most promising variant named as APPAmut9 (APPAmut8-D133E/R382I/S393I) was successfully identified. The *t*_1/2_ value of APPAmut9 at 65 °C was determined to be 256.7 min, showing an improvement of 9.1 min compared to that of APPAmut8 and approximately a 75.5-fold increase over that of APPAmut4. As shown in Fig. 3*I*, the *T*_50_ value for APPAmut9 exhibited an increase of 40.9 °C compared to that of APPAmut4, achieving a significant enhancement in thermostability with a temperature as high as 96.0 °C. Furthermore, the *T*_m_ of APPAmut9 experienced an elevation by 21.8 °C, reaching a value of 80.0 ± 1.1 °C. It is noteworthy that when exposed to boiling water at 100 °C, approximately 70% of the enzyme activity remained intact after a holding period of 5 min; even after an extended duration of 30 min, around 40% of initial activity was still retained (Fig. 3*J*). This phenomenon is exceptionally rare in the field of enzyme engineering research. Although the *T*_m_ value (75.7 °C) of *E*. *coli* phytase thermostable variant Phy9X was similar to that of APPAmut9, only 27% of initial activity remained after treatment at 85 °C for 10 min, indicating a greater potential application for APPAmut9 (37). The potential and stability of APPAmut9 is further demonstrated in Fig. 3*K*. Unlike APPAmut4, which underwent complete denaturation following treatment at 60 °C and 80 °C for 10 min, APPAmut9 exhibited stability even after exposure to 100 °C.

**Figure 4:**
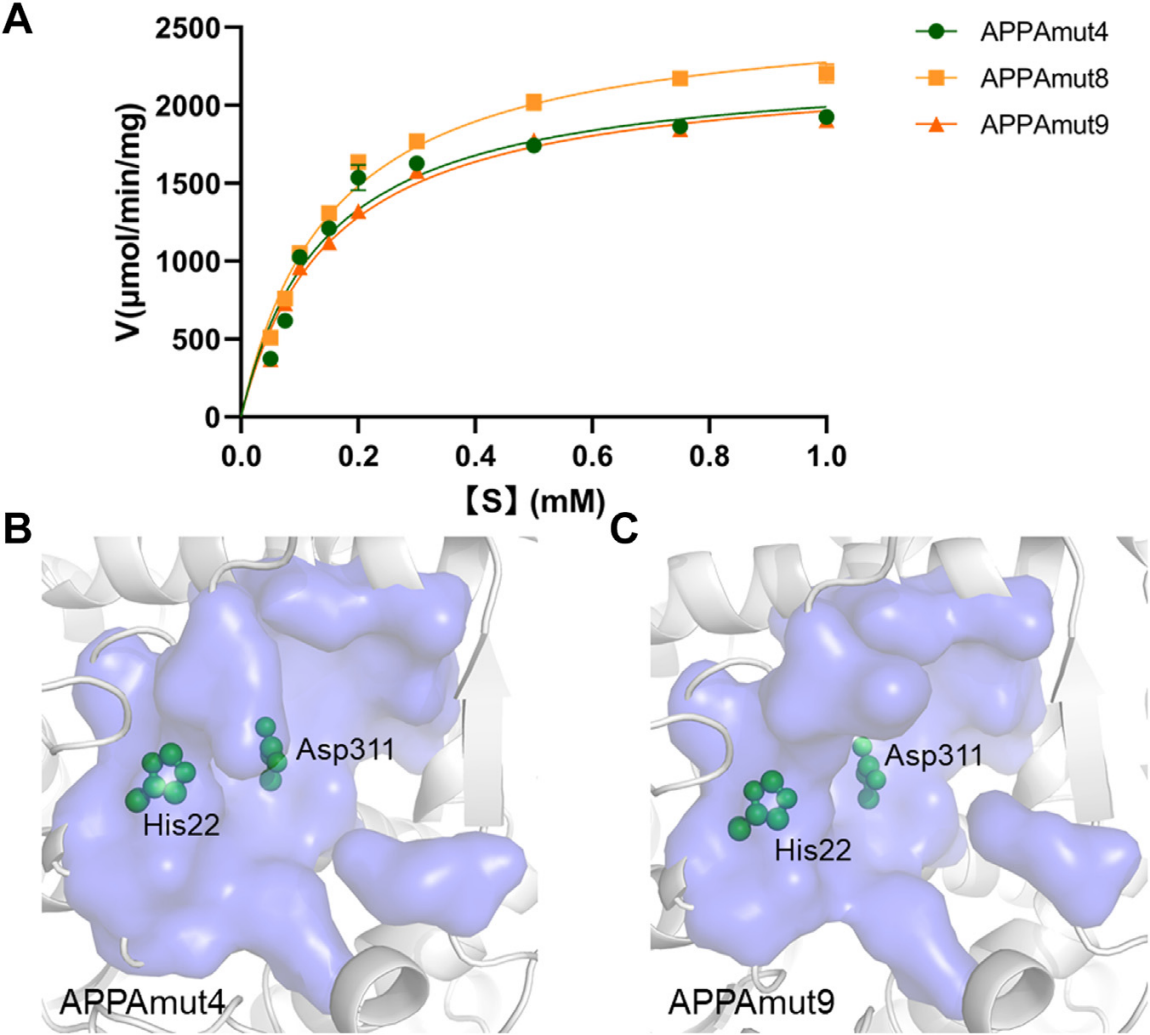
**The impact of mutations on phytase catalytic activity.***A*, the MichaelisMenten plots of APPAmut4, as well as its variant APPAmut8 and APPAmut9, were generated to determine the kinetic parameters under conditions of pH 5.5 and 37 °C. The catalytic pocket of APPAmut4 (*B*) and variant APPAmut9 (*C*) contains residues His22 and Asp311, which serve as the catalytic nucleophile and proton donor, respectively.

To evaluate the impact of mutations on phytase catalytic activity, the kinetic parameters of each variant using sodium phytate as a substrate were determined. All variants exhibited comparable catalytic activity to APPAmut4 (Fig. 4*A*). Particularly, APPAmut8 displayed a *K*_m_ value of 0.15 mM, which closely resembled that of APPAmut4. Moreover, its *k*_cat_ value was measured at 2000/s, indicating an approximate 15% increase compared to APPAmut4. Notably, the variant APPAmut9 exhibited a remarkable enhancement in thermostability without significant alterations in both *K*_m_ and *k*_cat_ values compared to those of APPAmut4, suggesting an unaltered catalytic efficiency. Consequently, by iteratively combining mutations, we successfully achieved a rational improvement of phytase’s thermostability to withstand temperatures up to 100 °C while maintaining its enzymatic activity.

### Structural interpretation of the enhanced thermostability in the variant APPAmut9

**Figure 5:**
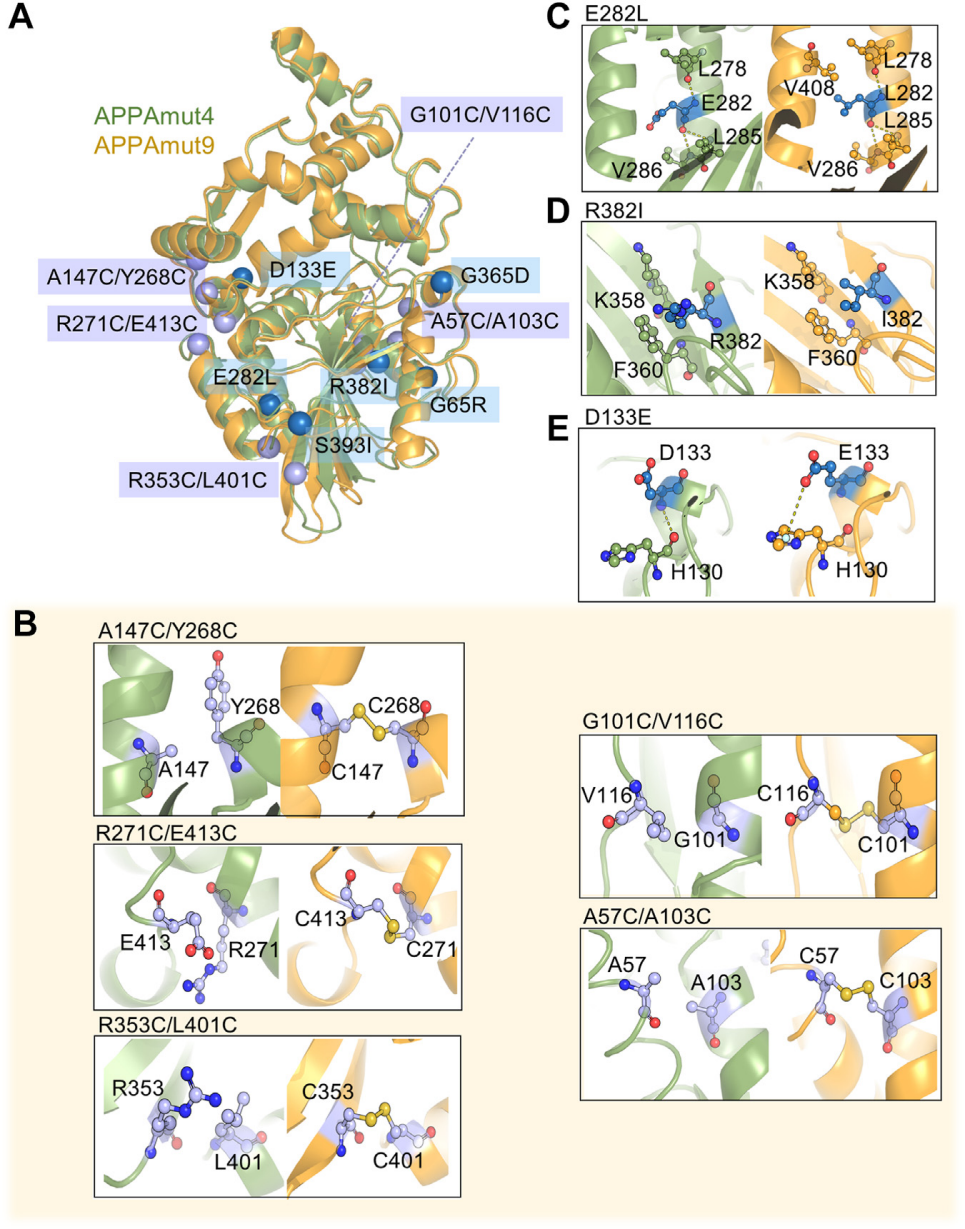
**Comparative analysis on the structural characteristics of phytase APPAmut4 and APPAmut9.***A*, the crystal structure of APPAmut4 and APPAmut9 is depicted, with the former shown in *green cartoon representation* and the latter in *orange*. The stabilizing mutations are indicated by their Cα atoms. *B*–*E*, the impact of the most effective mutations located within these structures is illustrated. Key residues in close proximity to the stabilizing mutations are represented as *purple* or *blue* ball-and-stick models.

In this study, a total of 37 beneficial mutation sites were identified in APPAmut4 to enhance its thermostability. Through combining and superimposing these mutations, the optimal variant APPAmut9 was selected, which exhibited a cumulative presence of additional five pairs of disulfide bonds and six single mutation sites distantly located from the catalytic pocket (Fig. 5*A*). To elucidate the impact of these 16 mutation sites on the catalytic function of APPAmut4, the crystal structure of APPAmut9 at a resolution of 1.77 Å was determined (PDB ID: 8XM2). Superimposition analysis revealed a structural similarity between APPAmut4 and APPAmut9, with only a slight deviation of 0.53 Å observed for 409 Cα atoms. Importantly, these mutation sites did not induce significant alterations in the catalytic pocket, thereby elucidating the absence of notable changes in catalytic efficiency between APPAmut9 and APPAmmut4 (Fig. 4, *B* and *C*).

The conformation of the four pairs of disulfide bonds in APPAmut4 is identical to that in APPAmut9, and these conserved disulfide bonds are also highly preserved in *E*. *coli* phytase to uphold structural stability (38). Through analysis of the mutation sites in the crystal structure, it was observed that the introduction of 10 cysteines effectively facilitated the formation of additional five pairs of disulfide bonds within the crystal structure of APPAmut9 (Fig. 5*B*, C147/C268, C271/C413, C353/C401, C101/C116, and C57/C103), each exhibiting distinct electron density. These covalent disulfide bonds establish crucial connections between diverse secondary structures such as α-helix and its surrounding α-helix, β-sheet, or loops, thereby ensuring robust structural stability of APPAmut9. In previous research, it was observed that the mutation of Thr83 to Arg resulted in the stabilization of the loop 74 to 87 regions on the protein surface through the formation of hydrogen bonds with Ala85 and Val86, as well as ionic interactions with Asp84 (39). In our study, we introduced four mutation sites (G101C/V116C and A57C/Y103C) to facilitate the formation of disulfide bonds between helix 95 to 108 and loop 108 to 131, as well as loop 37 to 59, respectively. This led to a significant stabilization of these two loop regions on the protein surface. The findings from both studies underscore the importance of the surface loop region in enhancing phytase stability.

Furthermore, the substitution of glutamate with leucine at position 282 in APPAmut4 results in the formation of additional hydrophobic interactions between Leu282 and Val408 on an adjacent α-helix, while maintaining three pairs of previously observed hydrogen bond interactions (Fig. 5*C*). Prior to the occurrence of the R382I mutation, Arg382 was involved in hydrophobic interactions with Lys358 and Phe360 (Fig. 5*D*). The substitution of Arg382 with Ile382 resulted in a reduction in the average B-factor value at this location (22.1 A^2^ *versus* 17.4 A^2^), leading to the stabilization of the β-sheet structure. With respect to the D133E mutation site, prior to the modification, hydrogen bonds were formed between the main chain N atom of Asp133 and the main chain O atom of His130. However, following the mutation, an anion–π electrostatic interaction was observed between the carboxyl O atom of Glu133 and imidazole ring of His130 due to elongation of side chain toward His130 (Fig. 5*E*). Given its limited dependence on distance and relatively low energy required for desolvation of histidine, this anion–π interaction can readily adapt to hydrophobic environments, thereby presenting a potential mechanism for thermal stabilization. In addition, the identified mutation sites G65R, G365D, and S393I are all situated in the solvent-exposed region, leading to enhanced interactions within the interconnecting loops and helices. The substitution of glycine with any other residue for G65R and G365D restricts the conformational space of the unfolded protein and reduces conformational entropy, similar to the effects observed with the introduction of disulfide bonds. Through comprehensive network optimization strategies, phytase APPAmut9 demonstrates exceptional thermostability with its remarkable nine pairs of disulfide bonds even under extreme heat conditions reaching temperatures as high as 100 °C.

## Discussion

Enhancing enzyme stability is crucial in expanding the application range of biotechnology and improving its adaptability. The development of high-temperature enzymes capable of withstanding temperatures up to 100 °C not only contributes to advancing enzyme engineering technology but also broadens the potential applications in various fields. To exemplify this concept further, we selected phytase due to its significant global market value amounting to 590 million US dollars (5). By thoroughly analyzing the crystal structure and implementing strategic approaches like introducing disulfide bonds, conducting energy calculations, and scrutinizing B-factors, rational thermostability design for APPAmut4 was successfully achieved in this study. Through iterative combination mutations, we successfully obtained a variant APPAmut9 with enhanced thermostability while maintaining its enzyme activity. Remarkably, this variant retains 70% of its activity even after exposure to the extreme temperature of 100 °C for 5 min, showcasing an exceptional level of thermal resilience rarely observed in the realm of enzyme engineering research.

Indeed, the thermostability of a phytase plays a crucial role in its suitability for industrial applications, and the creation of a novel thermostable phytase presents a formidable obstacle. Through the implementation of directed evolution and site-specific mutation techniques, successful engineering of *A*. *niger* N25 phytase phyA resulted in the identification of Q172R/K432R as the most heat-stable variant (20). This variant exhibited a *t*_1/2_ value of 12.8 min at 70 °C, approximately 2.1 times higher than that of the WT. To intelligently enhance the thermostability of *E*. *coli* phytase, a variant P56214 was successfully generated, exhibiting improved residual enzyme activity from 20% to 75% following brief exposure at temperatures as high as 90 °C (8). Building upon these achievements, our study has surpassed previous advancements by achieving even greater thermostability with variant APPAmut9. Notably, it demonstrated approximately 80% remaining enzyme activity after undergoing a 5-min treatment at 95 °C and maintained around 70% activity even when exposed to boiling water conditions at 100 °C for the same duration. These advancements fulfill the prerequisites for industrial-scale production and exhibit promising potential as an innovative phytase derivative for various applications.

The phytase was expressed in *Komagataella phaffii*, a well-known producer of diverse glycosylation patterns for different variants under specific growth conditions. In our previous study, we improved the kinetic stability of APPA by implementing *N*-glycosylation modification strategies (40). Interestingly, the *T*_m_ values of these variants remained unchanged, suggesting that the introduction of *N*-glycosylation modification enabled the variants to exhibit partial renaturation ability following exposure to high temperatures. To assess the potential impact of *N*-glycosylation modifications on the thermostability, we utilized the pierce glycoprotein staining kit (Real-Times) to detect *N*-glycosylation in APPAmut4 and its variants. Only the positive control (horseradish peroxidase) exhibited red band following staining with glycoprotein staining reagents, while APPAmut4, APPAmut5, APPAmut6, APPAmut7, APPAmut8, and APPAmut9 did not display red bands, indicating their nonglycosylated status. In contrast to variants generated through *N*-glycosylation modification strategy, it was observed that APPAmut9 showed a 40.9 °C increase in *T*_50_ value and a 21.8 °C increase in *T*_m_ value. These findings suggest that the enhanced thermostability of APPAmut9 is independent of *N*-glycosylation modification.

The expression level of APPAmut9 was indeed affected following the introduction of additional five disulfide bonds; however, this is not anticipated to pose a significant concern. Production bottlenecks were effectively addressed by employing bidirectional promoters for coexpression of folding chaperones (9, 41, 42). For example, coexpression of protein disulfide bond isomerase resulted in approximately a 12-fold increase in production of phytase ApV1 compared to expression without this folding catalyst and restored yields to levels comparable to those observed with the WT enzyme. From this perspective, there are no technical difficulties in efficiently expressing the superthermophilic phytase variant APPAmut9.

In conclusion, our study has successfully achieved thermostability of a phytase with resistance up to 100 °C, making it a promising candidate for industrial applications requiring high-temperature enzyme activity. These significant findings not only expand the repertoire of phytases available for industrial applications but also provide insight into the intricate relationship between enzyme structure and heat resistance, thereby establishing a solid theoretical foundation for future enzymatic modifications.

## Experimental procedures

### Materials, strains, and chemical agents

All chemical reagents utilized, except for those specifically indicated, were of analytical grade. Sodium phytate from Sigma-Aldrich served as the employed substrate. Chemically competent cells resistant to *Trans1*-T1 phage were obtained from TransGen Biotech. *K*. *phaffii* (formerly known as *Pichia pastoris* GS115) and vector pPICZαA were procured as the expression host and expression vector, respectively, from Novagen. The recombinant plasmid pPICZαA-*appa* employed in this study harbors the *Y*. *intermedia*–derived phytase gene (25).

### Site-specific mutagenesis

The Fast Mutagenesis Kit (TransGen) was utilized for site-directed mutagenesis using the pPICZαA-*appamut4* plasmid as a template. Primers were designed based on selected mutation sites and considering the frequency of amino acid codon usage in *K*. *phaffii*. Subsequently, *Dpn*I enzyme digestion was performed on the resulting PCR products to eliminate the original DNA template prior to individual transformation into chemically competent *Trans1*-T1 phage-resistant cells. Following this step, colony PCR was conducted to amplify the target genes, and positive clones underwent sequencing for validation of plasmid accuracy.

### Induced expression and purification of phytase

The strains carrying the target genes were inoculated into a yeast extract peptone dextrose medium and incubated at 30 °C for 48 h to obtain a seed solution. Subsequently, the strains were transferred and cultured at a speed of 200 rpm at 30 °C for another period of 48 h in 1 L Erlenmeyer flasks containing 400 ml buffered glycerol-complex medium. The culture was then centrifuged at a speed of 4500 rpm for 5 min, and the supernatant was discarded. Following this step, a 200 ml buffered methanol-complex medium was added to induce protein expression at 30 °C for 48 h (with methanol supplementation every 12 h), followed by centrifugation to collect the desired target protein in the supernatant.

The fermentation supernatant was concentrated using a 10 kDa molecular weight cut-off membrane package to remove impurities such as pigments and salt ions. Subsequently, the concentrated fermentation solution underwent dialysis with a 3 kDa molecular weight cut-off, utilizing a dialysate consisting of 20 mM Tris–HCl buffer at pH 8.0 (buffer A). The initial purification step involved HiTrap Q HP column fast protein liquid chromatography, which was pre-equilibrated with buffer A. To elute the enzyme, a linear gradient of buffer B (buffer A containing 1 M NaCl) was employed. Each fraction collected during the gradient elution process was selected based on its enzymatic activity toward the sodium phytate substrate. Further purification of the enzyme was subsequently achieved by employing a Superdex-75 pg column. Finally, analysis of the purified enzymes was conducted using SDS-PAGE.

### Crystal growth, diffraction, and structural analysis

The Hampton Research kit was utilized to conduct a comprehensive screening of crystallization conditions for phytase. Crystals were grown in 24-well plates using the hanging-drop vapor diffusion method, ensuring efficient screening of crystal conditions. Each well was filled with 500 μl of reservoir solution, and a cover glass containing a mixture of 2 μl protein sample and 2 μl reservoir solution was gently placed on top and sealed with specialized adhesive. Subsequently, the crystals were cultured at a temperature of 16 °C under microscopic observation to monitor their growth. Well-developed crystals were carefully selected based on size and handpicked using a specialized loop and then transferred to a cryo-buffer (reservoir solution +25% glycerin). These meticulously chosen crystals were promptly preserved in liquid nitrogen for further analysis and experimentation. Crystallographic diffraction experiments took place at beamline BL10U2 of the Shanghai Synchrotron Radiation Facility. Subsequent data processing was carried out using Phenix software (43). The molecular displacement methodology employed the crystal structure of *Yersinia kristensenii* phytase (PDB ID: 4ARV, 88% identity) as a template for analysis.

### Prediction of mutations aided by computational tools

The disulfide bond engineering design for APPAmut4 aimed at enhancing its thermostability was conducted using Disulfide by Design (http://cptweb.cpt.wayne.edu/DbD2/) and Discovery Studio (http://www.accelrys.com/) analysis. These two algorithms were combined to calculate all residue pairs capable of forming disulfide bonds, while ensuring the SG−SG atom pairs maintained a standard distance range (1.0–3.0 Å) and the cysteine side chain had a dihedral angle range (75.0–135.0°). Furthermore, the impact of mutations on various parameters such as energy, adverse contacts, residue depth, and changes in residue volume before and after mutation was evaluated. The changes in folding free energy (ΔΔG) caused by saturation mutation of all amino acid residues of APPAmut4 were calculated using FoldX (44), CUPSAT (45), and DeepDDG (46), respectively. FoldX assesses the impact of mutations on protein stability, folding, and dynamics by comparing variant folding free energy with WT folding free energy (44). CUPSAT predicts alterations in protein stability after mutations based on atomic and twist angle potential within a specific structural environment (45). DeepDDG utilizes neural networks to predict changes in protein stability caused by mutations (46). HotSpot Wizard 3.0 (https://loschmidt.chemi.muni.cz/hotspotwizard/) was employed to automatically identify hot spots in semirational protein design aiming at enhancing protein stability based on the structural design module (47). The B-factor value for each residue in the phytase crystal structure APPAmut4 is determined by averaging the B-factor values of all constituent atoms using the B-FITTER tool (48), as an amino acid comprises multiple atoms.

### Determination of phytase activity

The enzyme solution was blended with a pH-adjusted acetate-acetic acid buffer (0.1 M, pH 5.5), supplemented with bovine serum albumin (0.05%) and Triton X-100 (0.05%), before undergoing dilution steps. Afterward, an aliquot of the diluted enzyme solution (100 μl) was incubated with sodium phytate substrate (5 mM, 900 μl) at precisely controlled conditions of temperature (37 °C) for a defined period of time lasting 10 min. To terminate the reaction, 1 ml of a trichloroacetic acid solution with a concentration of 10% w/v was added. Subsequently, coloring reagent C—comprising ammonium molybdate tetrahydrate (1% w/v), sulfuric acid (3.2% v/v), and ferrous sulfate heptahydrate (7.32% w/v)—was introduced for a reaction period of 5 min before measuring the absorption at a wavelength of 700 nm. In contrast, trichloroacetic acid was initially added to the substrate as a control. To ensure accuracy and reproducibility in determining enzyme activity (U), three parallel reactions were conducted for each trial. Enzyme activity is defined as the quantity of enzyme required to release 1 μmol of inorganic phosphorus per minute under standardized conditions set at pH 5.5 and 37 °C.

### Stability and kinetic parameters

The optimal temperature for phytase activity was determined by assessing its performance at pH 5.5 across a temperature range of 30 to 70 °C (at 5 °C intervals). Subsequently, the half-life *t*_1/2_ of phytase at a temperature of 65 °C was determined. The enzyme solution was appropriately diluted and incubated at 65 °C for various durations (0, 2, 5, 10, 15, 30, 60, and 120 min), followed by measurement of the corresponding activity under standardized conditions. The initial activity served as a reference point (100%). Furthermore, the enzyme solution was incubated for 30 min within a temperature range of 45 to 75 °C. Subsequent measurements under standardized conditions were utilized to calculate the remaining enzyme activities at different temperatures relative to unincubated samples with an assigned value of 100%. The specific temperature *T*_50_ value represents the temperature at which the enzyme retains 50% of its maximum activity.

Phytase activity was assessed at 37 °C in a water bath and pH 5.5, using varying concentrations of sodium phytate (0.05–1.00 mM) as the substrate. Subsequently, data analysis was performed using GraphPad Prism software (https://www.graphpad-prism.cn), with the Michaelis equation fitted to determine *K*_m_ and *k*_cat_ values. The experiments were conducted in triplicate and repeated three times for each experiment.

### *T*_m_ value measurement

The thermal denaturation temperature of phytase was determined using a Chirascan circular dichroism spectrometer from Applied Photophysics Ltd. A 300 μl solution containing 50 μg/ml of the protein in 10 mM acetoacetate-sodium acetate buffer at pH 5.5 was loaded into a 1 mm path quartz cuvette, and its far-UV spectral changes between 190 and 260 nm were measured at various temperatures. The experiment was conducted over the range of 25 to 100 °C with a temperature ramp rate of 1 °C/min and a step size of 1 nm, with each scan repeated three times. After background subtraction using CDNN software, the ellipticity values at either 208 or 222 nm were obtained, from which the folding fraction (α) was calculated using formula (1, 49, 50). The *T*_m_ value for phytase was determined by fitting the data using GraphPad Prism version 8.0 software. *T*_m_ corresponds to the temperature at α = 0.5.

(1) α=(θt−θU)/(θF−θU)

where *θ*_t_ represents the observed ellipticity at 25 to 100 °C, *θ*_U_ denotes the ellipticity of the native form, and *θ*_F_ indicates the ellipticity of the folded form.

## Data availability

All data needed to evaluate the conclusions in the article are present in the article.

Additional data is available from authors upon request.

## Conflict of interest

The authors declare that they have no conflicts interest with the contents of this article.
